# The Chicago Veterinary College

**Published:** 1901-04

**Authors:** 


### THE CHICAGO VETERINARY COLLEGE.

The commencement exercises of the eighteenth session of the Chicago Veterinary College were held in the College Auditorium on Wednesday, March 27th, last. The following gentlemen received their diplomas and the degree of Doctor of Comparative Medicine: E. M. Bronson, Indianapolis, Ind.; O. A. Kyle, Colfax, Ill.; C. G. Jennings, Marseilles, Ill.; W. R. Michael, St. Jacob, Ill.; James C. Myers, Chicago, Ill.; W. R. Pick, Lodi, Wis.; I. D. Reynierson, Jamestown, Ind.; J. O. Simcoke, Stewart, Ia.; and E. B. Ward, Perry Mo.

Dr. Michael distinguished himself by winning the gold medal for the highest general average, also the prizes in theory and practice and anatomy. Prof. A. H. Baker delivered the doctorate address, referring to the present bright prospects of the veterinary profession evinced by the extraordinary large demands for veterinary surgeons all over the country and large attendance of students, also giving the graduating class some valuable advice and wishing them godspeed in their future career.

